# Study on the synthesis and biological activity of kojic acid triazol thiosemicarbazide Schiff base derivatives

**DOI:** 10.1080/14756366.2025.2475071

**Published:** 2025-04-08

**Authors:** Yayuan Luo, Zhiyong Peng, Junyuan Tang, Dahan Wang, Sheng Tao, Jinbing Liu

**Affiliations:** a School of Food and Chemical Engineering, Shaoyang University, Shaoyang, People’s Republic of China; b Chengda Pharmaceuticals Co., Ltd., Jiaxing, People’s Republic of China

**Keywords:** Kojic acid triazol, thiosemicarbazide Schiff base, tyrosinase, antioxidant, anti-browning

## Abstract

A series of kojic acid triazol thiosemicarbazide Schiff base derivatives were designed and synthesised. Evaluation on the inhibition of tyrosinase activity showed that these compounds possessed potent inhibit tyrosinase activity, and the compound **6w** (IC_50_ = 0.94 μM) exhibited the best inhibitory effect. Preliminary structure–activity relationships indicate that steric hindrance, halogen atom radius, and electron donating ability of functional groups have some impact on the inhibition of tyrosinase activity. Inhibition mechanism showed that compound **6w** is a non-competitive mixed inhibitor, and this result was further confirmed by molecular docking. The fluorescence quenching mode of compound **6w** is dynamic quenching, and interacts with tyrosinase by changing the amide structure of tyrosinase. Compound **6w** has some anti-browning effect. Compound **6p** had the strongest DPPH radical scavenging activity (IC_50_ = 10.53 ± 0.014 μM), and compound **6w** showed the best ABTS scavenging activity (IC_50_ = 3.03 ± 0.009 μM).

## Introduction

Tyrosinase, also known as polyphenol oxidase or catechol oxidase, is a complex multi-subunit copper-containing metalloenzyme that is widely present in the human body, microorganisms, animals, and plants^1–4^. It is the key rate limiting enzyme that regulates melanin production^5^. Overexpression of tyrosinase can cause a series of skin diseases related to pigmentation, such as freckles, melasma, age spots, melanoma, etc.^6^ Tyrosinase is also related to insect moulting, wound healing, Parkinson’s disease^7^^,^^8^, and is an important factor in causing enzymatic browning of fruits and vegetables^9^^,^^10^. Therefore, tyrosinase inhibitors have potential applications in pharmaceutical and cosmetic products, and also can be used in food industry. Several natural and synthetic tyrosinase inhibitors have been reported to date^11–14^, but due to their limitations concerning cytotoxicity, selectivity, and stability^15^^,^^16^. Hence, developing efficient and non-toxic tyrosinase inhibitors is of great importance for the food, medicine, and agriculture fields.

Oxidative stress, an imbalance between antioxidant systems and the production of oxidants, mainly induced by reactive oxygen species (ROS), leads to oxidative degradation of biomolecules^17^^,^^18^. An appropriate amount of ROS can participate in regulating cell signalling pathways, inducing and promoting mitotic reaction, maintaining redox homeostasis and fighting infection factors^19^. Excessive ROS can damage DNA, protein, nucleic acid, and lipid, and can also cause excessive oxidative reaction, leading to human ageing and a variety of chronic diseases, such as cancer, cardiovascular disease, hypertension, atherosclerosis, diabetes, etc.^20^ Thus, the antioxidant effect in cosmetics such as whitening agents is highly considered.

Kojic acid, a natural product bearing the 3-hydroxy-4-pyranone scaffold, is a well-known antityrosinase agent. Kojic acid has a wide range of pharmacological profiles, such as antioxidant, antibacterial, anti-tumour, anti-inflammatory, and antiproliferative activities^21^^,^^22^. Kojic acid has been used mainly as a whitening agent in cosmetics^23^. However, kojic acid has the defects of easy decomposition and side effects^24^. Based on this, domestic and foreign scholars have synthesised a large number of kojic acid derivatives based on its structure and evaluated their tyrosinase inhibitory activity and antioxidant activity^25–27^. Experiments have shown that these derivatives exhibit higher stability and lower toxicity than kojic acid. Triazole is a class of heterocyclic compounds with an extensive range of applications in the fields of pharmaceuticals, biology, agriculture, sensing, and material chemistry^28^^,^^29^. The 1,2,3-triazole derivatives have a wide range of pharmacological activities, including antibacterial, antiviral, anti-tuberculosis, antidepressant, anticonvulsant, anti-inflammatory, antioxidant, anticancer, antioxidant, and enzyme inhibitory activities^30^^,^^31^. In order to obtain new tyrosinase inhibitors and antioxidants, based on previous research work, the principle of active fragment combination was adopted to combine kojic acid and triazole structures into one molecule, investigating enzyme inhibition activity and antioxidant activity, and exploring enzyme inhibition mechanism.

## Materials and methods

### Instruments and reagents

All the reactions described below were monitored by thin layer chromatography (TLC), which was performed using Merck precoated 60F254 plates (Rahway, NJ). Melting points were measured on WRS-1B melting point apparatus and are uncorrected.^1^H NMR and ^13^C NMR spectra were recorded on a JNM-ECZ400S/L1 400 MHz at 25 °C in DMSO-*d_6_* using tetramethyl silane (TMS) as an internal standard. Coupling constant (*J*) and chemical shift values were measured in hertz (Hz) and parts per million (ppm), respectively. The following abbreviations are used for ^1^H NMR: s (singlet), d (doublet), dd (doublet of doublets), t (triplet), and m (multiplet). FT-IR spectra were obtained on a Nicolet-iS5 spectrophotometer (KBr disks). Mass spectrometric (MS) data are reported in *m/z* using the Q Exactive Focus. UV-2600 spectrophotometer (Shimadzu Corporation, Tokyo, Japan) was used to record UV spectra. The fluorescence spectra of the selected compound were inspected on Cary Eclipse G9800A fluorescence spectrophotometer (Agilent Technologies Co., Santa Clara, CA). The colourimetric measurements were carried out by a CR-400 Minolta chronometer instrument (Konica Minolta, Osaka, Japan). The model of ultrasonic cleaning machine is SB-5200DT (Ningbo Xinzhi Biotechnology Co., Ltd., Ningbo, China). Purity for target compounds was measured by high-performance liquid chromatography (HPLC) with a Thermo Scientific UltiMate 3000 (Waltham, MA). Tyrosinase, l-3,4-dihydroxyphenylalanine (l-DOPA), 1,1-diphenyl-2-picrylhydrazyl radical 2,2-diphenyl-1-(2,4,6-trinitrophenyl)hydrazyl (DPPH), 2,2′-azino-bis(3-ethylbenzo-thiazoline-6-sulfonic acid) (ABTS), and kojic acid were purchased from Sigma-Aldrich Chemical Co. (Shanghai, China). Other chemicals were obtained from commercial suppliers and used without further purification.

### Synthesis and characterisation

See Scheme 1.

**Scheme 1.**
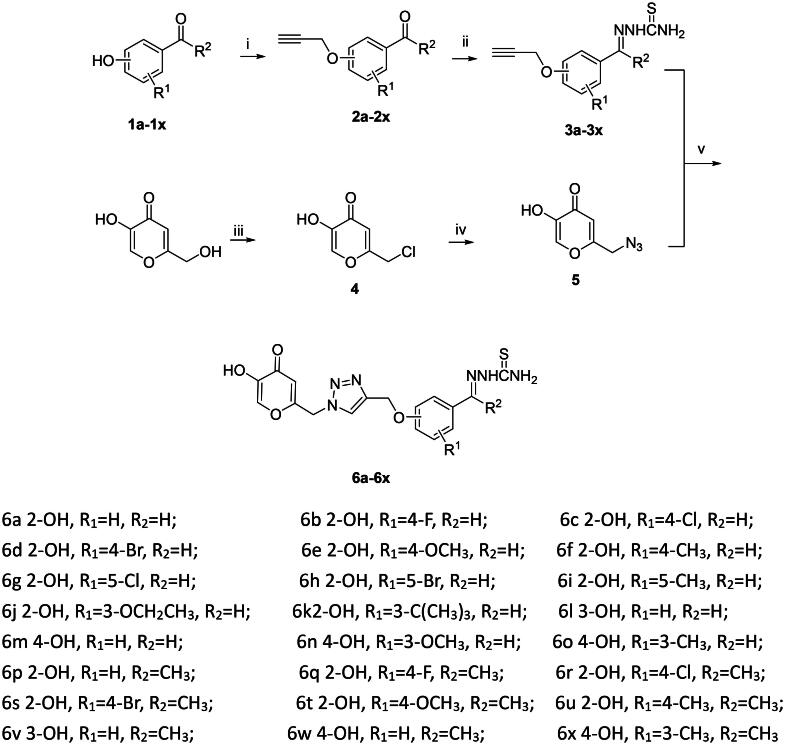
Synthesis pathway of kojic acid azole aminothiourea Schiff base derivatives. Reagents and conditions: (I) propargyl bromide, potassium carbonate, acetone, RT; (II) thiosemicarbazide, anhydrous ethanol, glacial acetic acid, RT; (III) thionyl chloride, DCM, RT; (IV) sodium azide, DMF, RT; (V) THF, Copper sulphate pentahydrate, sodium ascorbate, reflux.

### Method for the synthesis of 2-(azidomethyl)-5-hydroxy-4H-pyran-4-one (5)

To a rapidly stirring suspension of kojic acid (14.21 g, 100 mmol) in CH_2_Cl_2_ (100 ml) at room temperature was added thionyl chloride (22 ml, 300 mmol) by dropwise over the course of 30 min. Then, the reaction was stirred for 6–8 h at room temperature. The reaction process was monitored by TLC (petroleum ether:ethyl acetate = 1:1 (v/v)). After the reaction was completed, the precipitates were filtered and washed with acetone to afford kojic chloride as a white solid in 85% (13.6 g) yield.

A mixture of kojic chloride 8.03 g (50.0 mmol), sodium azide 3.25 g (50.0 mmol) in 60 ml DMF was stirred for 8 h at room temperature. The reaction was stirred at room temperature. The reaction process was monitored by TLC (petroleum ether:ethyl acetate = 1:1 (v/v)). After the reaction was completed, water (60 ml) was added to the reaction mixture. The mixture was extracted with ethyl acetate (3 × 50 ml), the organic phase was washed three times with water, and dried over anhydrous magnesium sulphate. The solvent was removed by vacuum evaporation. 2-(Azidomethyl)-5-hydroxy-4H-pyran-4-one was obtained as a yellow solid in 61.7% (5.15 g) yield.

### General method for the synthesis of propargyl ether thiosemicarbazide Schiff base (3a–3x)

Different substituted hydroxyl aromatic aldehydes or ketones (10 mmol) and propargyl bromide 2.38 g (20 mmol) were placed in a 50 ml reaction flask, and then dried potassium carbonate 4.15 g (30 mmol) and acetone 30 ml were added to the mixture. The reaction mixture was stirred at room temperature for about 5 h, and the reaction progress was detected by TLC (petroleum ether:ethyl acetate = 5:1). The reaction was completed; reaction mixture was filtrated. The filter cake was washed with acetone. Filtrate was removed the solvent by vacuum evaporation and the residue was recrystallised with ethanol to obtain the alkyne ether compound.

The alkyne ether compound (10 mmol) and thiosemicarbazide 0.91 g (10 mmol) were placed in a 50 ml reaction flask, and then 20 ml of anhydrous ethanol and 10 drops of glacial acetic acid were added. The reaction mixture was stirred at room temperature for 4 h, and the reaction progress was detected by TLC (petroleum ether:ethyl acetate = 3:1). After the reaction was complete, the precipitates were filtered and recrystallised with ethanol to obtain the propargyl ether thiosemicarbazide Schiff base compounds with yield of 60–80%.

### General method for the synthesis of kojic acid triazol thiosemicarbazide Schiff base (6a–6x)

The 2-(azidomethyl)-5-hydroxy-4H-pyran-4-one 0.50 g (2 mmol) and propargyl ether thiosemicarbazide Schiff base (2 mmol) were placed in a 25 ml reaction flask, and 8 ml tetrahydrofuran was added. The reaction mixture was stirred at room temperature. Copper sulphate pentahydrate 0.040 g (0.16 mmol) was dissolved in 8 ml of water; 0.10 g (0.5 mmol) sodium ascorbate was added to the solution and stirred to dissolve. The mixture was added to the above reaction flask. Then, the reaction mixture was heated to reflux. The reaction progress was detected by TLC (petroleum ether:ethyl acetate = 1:1). After the reaction was complete, appropriate amount of water was added to the reaction mixture, and then extracted three times with ethyl acetate. The organic phase was combined and dried with anhydrous sodium sulphate. The solvent was removed by vacuum evaporation. The residue was purified by column chromatography to obtain target compound.

#### (2-((1-((5-Hydroxy-4-oxo-4H-pyran-2-yl)methyl)-1H-1,2,3-triazol-4-yl)methoxy)benzylidene)hydrazinecarbothioamide (6a)

Grey solid, yield 37.22%, m.p. 220.8–223.8 °C, 98% purity. IR (KBr, *ν*/cm^−1^): 3389, 3136, 1623, 1600, 1532, 1211, 1090, 750. ^1^H NMR (400 MHz, DMSO-*d_6_*) *δ* 11.41 (s, 1H, –NH), 8.37 (s, 1H, ═CH), 8.30 (s, 1H, ═CH), 8.17–8.14 (m, 1H, ═CH), 8.14–8.12 (m, 1H, Ph-H), 8.12–8.10 (m, 1H, Ph-H), 8.01 (d, *J* = 17.1 Hz, 2H, –NH_2_), 7.37 (s, 1H, Ph-H), 7.05–6.99 (m, 1H, Ph-H), 6.40 (s, 1H, ═CH), 5.62 (s, 2H, –CH_2_), 5.26 (s, 2H, –CH_2_). ^13^C NMR (101 MHz, DMSO-*d_6_*) *δ* 174.11, 161.00, 160.58, 157.02, 156.36, 146.54, 143.61, 140.53, 138.53, 131.68, 126.68, 125.77, 123.12, 121.57, 113.65, 113.56, 62.28, 50.50. HRMS (ESI) *m/z*: [M + H]^+^: 401.1027; found: 401.1023.

#### 2-(4-Fluoro-2-((1-((5-hydroxy-4-oxo-4H-pyran-2-yl)methyl)-1H-1,2,3-triazol-4-yl)methoxy)benzylidene)hydrazinecarbothioamide (6b)

Grey solid, yield 30.12%, m.p. 162.9–164.5 °C, 98% purity. IR (KBr, *ν*/cm^−1^): 3366, 3184, 1653, 1599, 1540, 1165, 1050, 836. ^1^H NMR (400 MHz, DMSO-*d_6_*) *δ* 11.39 (s, 1H, –NH), 9.33 (s, 1H, ═CH), 8.40 (s, 1H, ═CH), 8.34 (s, 1H, ═CH), 8.23–8.11 (m, 2H, –NH_2_), 8.08 (s, 1H, Ph-H), 7.23 (d, *J* = 10.9 Hz, 1H, Ph-H), 6.86 (t, *J* = 8.3 Hz, 1H, Ph-H), 6.45 (s, 1H, ═CH), 5.66 (s, 2H, –CH_2_), 5.29 (s, 2H, –CH_2_). ^13^C NMR (101 MHz, DMSO-*d_6_*) *δ* 174.22, 165.79, 163.33, 161.07, 158.43, 158.32, 146.65, 140.64, 137.94, 128.54, 128.44, 126.10, 119.79, 113.69, 108.72, 108.51, 101.93, 101.67, 62.68, 50.61. HRMS (ESI) *m/z*: [M + H]^+^: 419.0932; found: 419.0929.

#### 2-(4-Chloro-2-((1-((5-hydroxy-4-oxo-4H-pyran-2-yl)methyl)-1H-1,2,3-triazol-4-yl)methoxy)benzylidene)hydrazinecarbothioamide (6c)

Brown solid, yield 31.45%, m.p. 175.4–177.5 °C, 97% purity. IR (KBr, *ν*/cm^−1^): 3410, 3217, 1648, 1592, 1533, 1124, 1056, 867. ^1^H NMR (400 MHz, DMSO-*d_6_*) *δ* 11.41 (s, 1H, –NH), 8.37 (s, 1H, ═CH), 8.30 (s, 1H, ═CH), 8.16 (s, 1H, ═CH), 8.12 (d, *J* = 8.4 Hz, 1H, Ph-H), 8.01 (d, *J* = 17.1 Hz, 2H, –NH_2_), 7.37 (s, 1H, Ph-H), 7.05–6.99 (m, 1H, Ph-H), 6.40 (s, 1H, ═CH), 5.62 (s, 2H, –CH_2_), 5.26 (s, 2H, –CH_2_). ^13^C NMR (101 MHz, DMSO-*d_6_*) *δ* 160.95, 157.48, 146.51, 143.12, 140.55, 137.50, 135.77, 127.94, 125.98, 122.18, 121.71, 114.08, 113.60, 62.58, 60.28, 50.51, 14.53. HRMS (ESI) *m/z*: [M + H]^+^: 435.0637; found: 435.0632.

#### 2-(4-Bromo-2-((1-((5-hydroxy-4-oxo-4H-pyran-2-yl)methyl)-1H-1,2,3-triazol-4-yl)methoxy)benzylidene)hydrazinecarbothioamide (6d)

Brown solid, yield 31.98%, m.p. 162.9–164.5 °C, 97% purity. IR (KBr, *ν*/cm^−1^): 3440, 3226, 1652, 1619, 1516, 1149, 1071, 853. ^1^H NMR (400 MHz, DMSO-*d_6_*) *δ* 11.52 (s, 1H, –NH), 9.34 (s, 1H, ═CH), 8.38 (s, 1H, ═CH), 8.34 (s, 1H, ═CH), 8.07 (d, *J* = 4.3 Hz, 2H, –NH_2_), 8.06 (s, 1H, Ph-H), 7.53 (s, 1H, Ph-H), 7.19 (d, *J* = 8.1 Hz, 1H, Ph-H), 6.45 (s, 1H, ═CH), 5.65 (s, 2H, –CH_2_), 5.31 (s, 2H, –CH_2_). ^13^C NMR (101 MHz, DMSO-*d_6_*) *δ* 159.37, 148.85, 146.12, 137.32, 132.51, 130.71, 122.66, 120.90, 119.72, 118.05, 113.57, 110.96, 106.35, 74.08, 70.16, 60.49, 40.56. HRMS (ESI) *m/z*: [M + H]^+^: 479.0132; found: 479.0127.

#### 2-(2-((1-((5-Hydroxy-4-oxo-4H-pyran-2-yl)methyl)-1H-1,2,3-triazol-4-yl)methoxy)-4-methoxybenzylidene)hydrazinecarbothioamide (6e)

Grey solid, yield 35.78%, m.p. 133.9–135.9 °C, 99% purity. IR (KBr, *ν*/cm^−1^): 3380, 3164, 1653, 1597, 1549, 1163, 1031, 870. ^1^H NMR (400 MHz, DMSO-*d_6_*) *δ* 11.36 (s, 1H, –NH), 9.36 (s, 1H, ═CH), 8.39 (d, *J* = 13.1 Hz, 1H, ═CH), 8.10 (s, 1H, ═CH), 8.03 (d, *J* = 30.0 Hz, 1H, ═CH), 6.86 (s, 1H, Ph-H), 6.63 (s, 1H, Ph-H), 6.47 (s, 1H, Ph-H), 5.68 (s, 2H, –CH_2_), 5.31 (s, 2H, –CH_2_), 3.85 (s, 3H, –OCH_3_). ^13^C NMR (101 MHz, DMSO-*d_6_*) *δ* 159.38, 146.80, 132.50, 131.42, 120.74, 110.92, 106.05, 99.98, 74.07, 69.93, 68.28, 64.85, 60.49, 49.09, 45.08, 40.55, 37.07, 31.72. HRMS (ESI) *m/z*: [M + H]^+^: 431.1132; found: 431.1130.

#### 2-(2-((1-((5-Hydroxy-4-oxo-4H-pyran-2-yl)methyl)-1H-1,2,3-triazol-4-yl)methoxy)-4-methylbenzylidene)hydrazinecarbothioamide (6f)

Brown solid, yield 40.57%, m.p. 120.7–123.1 °C, 99% purity. IR (KBr, *ν*/cm^−1^): 3410, 3265, 1645, 1609, 1456, 1219, 1074, 885, 789. ^1^H NMR (400 MHz, DMSO-*d_6_*) *δ* 10.08 (s, 1H, –NH–), 8.47 (s, 1H, ═CH), 7.69 (s, 1H, ═CH), 7.46 (s, 1H, ═CH), 7.39 (d, *J* = 6.3 Hz, 1H, Ph-H), 6.68 (s, 1H, Ph-H), 6.45 (d, *J* = 6.3 Hz, 1H, Ph-H), 6.14 (s, 1H, ═CH), 5.52 (s, 2H, –CH_2_), 5.19 (s, 2H, –CH_2_), 2.87 (s, 3H, –CH_3_). ^13^C NMR (101 MHz, DMSO-*d_6_*) *δ* 159.37, 148.90, 145.69, 137.32, 135.01, 133.67, 132.50, 131.22, 121.30, 120.67, 118.00, 116.35, 111.40, 110.90, 74.07, 69.84, 60.48, 40.55, 37.62. HRMS (ESI) *m/z*: [M + H]^+^: 415.1184; found: 415.1183.

#### 2-(5-Chloro-2-((1-((5-hydroxy-4-oxo-4H-pyran-2-yl)methyl)-1H-1,2,3-triazol-4-yl)methoxy)benzylidene)hydrazinecarbothioamide (6g)

Yellow solid, yield 35.24%, m.p. 182.3–183.9 °C, 97% purity. IR (KBr, *ν*/cm^−1^): 3410, 3217, 1660, 1595, 1533, 1129, 1100, 859. ^1^H NMR (400 MHz, DMSO-*d_6_*) *δ* 11.43 (s, 1H, –NH), 8.40 (s, 1H, –OH), 8.32 (s, 1H, ═CH), 8.21 (d, *J* = 2.4 Hz, 1H, ═CH), 8.18 (s, 1H, ═CH), 8.06 (s, 1H, Ph-H), 7.92 (s, 2H, –NH_2_), 7.37 (m, *J* = 8.9, 2.4 Hz, 1H, Ph-H), 7.27 (d, *J* = 9.3 Hz, 1H, Ph-H), 6.41 (s, 1H, ═CH), 5.62 (s, 2H, –CH_2_), 5.23 (s, 2H, –CH_2_). ^13^C NMR (101 MHz, DMSO-*d_6_*) *δ* 178.44, 174.14, 162.87, 160.96, 155.65, 146.50, 143.24, 140.53, 125.95, 113.62, 62.63, 60.25, 50.47, 36.29, 31.26, 21.23, 14.54. HRMS (ESI) *m/z*: [M + H]^+^: 435.0637; found: 435.0632.

#### 2-(5-Bromo-2-((1-((5-hydroxy-4-oxo-4H-pyran-2-yl)methyl)-1H-1,2,3-triazol-4-yl)methoxy)benzylidene)hydrazinecarbothioamide (6h)

Yellow solid, yield 35.71%, m.p. 274.8–276.1 °C, 99% purity. IR (KBr, *ν*/cm^−1^): 3473, 3154, 1657, 1582, 1540, 1139, 1093, 839. ^1^H NMR (400 MHz, DMSO-*d_6_*) *δ* 11.46 (s, 1H, –NH), 9.33 (s, 1H, ═CH), 8.38 (s, 1H, ═CH), 8.34 (s, 2H, –NH_2_), 8.07 (s, 1H, ═CH), 7.95 (s, 1H, Ph-H), 7.52 (d, *J* = 8.2 Hz, 1H, Ph-H), 7.25 (d, *J* = 8.7 Hz, 1H, Ph-H), 6.44 (s, 1H, ═CH), 5.65 (s, 2H, –CH_2_), 5.27 (s, 2H, –CH_2_). ^13^C NMR (101 MHz, DMSO-*d_6_*) *δ* 159.37, 150.33, 148.85, 146.27, 144.97, 137.31, 134.73, 132.51, 129.93, 127.13, 123.06, 120.83, 120.35, 112.87, 111.20, 110.94, 70.10, 60.49, 49.10, 45.09. HRMS (ESI) *m/z*: [M + H]^+^: 479.0132; found: 479.0131.

#### 2-(2-((1-((5-Hydroxy-4-oxo-4H-pyran-2-yl)methyl)-1H-1,2,3-triazol-4-yl)methoxy)-5-methylbenzylidene)hydrazinecarbothioamide (6i)

Yellow solid, yield 36.83%, m.p. 283.3–285.1 °C, 97% purity. IR (KBr, *ν*/cm^−1^): 3364, 3148, 1659, 1534, 1549, 1159, 1079, 830. ^1^H NMR (400 MHz, DMSO-*d_6_*) *δ* 11.38 (s, 1H, –NH), 9.33 (s, 1H, ═CH), 8.37 (d, *J* = 15.7 Hz, 2H, –NH_2_), 8.17 (s, 1H, ═CH), 8.08 (s, 1H, ═CH), 7.95 (s, 1H, Ph-H), 7.19 (d, *J* = 8.0 Hz, 1H, Ph-H), 7.15 (d, *J* = 8.5 Hz, 1H, Ph-H), 6.43 (s, 1H, ═CH), 5.65 (s, 2H, –CH_2_), 5.22 (s, 2H, –CH_2_), 2.27 (s, 3H, –CH_3_). ^13^C NMR (101 MHz, DMSO-*d_6_*) *δ* 159.37, 148.89, 144.16, 137.31, 135.05, 132.51, 131.05, 125.83, 124.47, 121.53, 120.67, 118.31, 111.04, 110.91, 69.99, 60.47, 48.47, 36.51. HRMS (ESI) *m/z*: [M + H]^+^: 415.1183; found: 415.1178.

#### 2-(3-Ethoxy-2-((1-((5-hydroxy-4-oxo-4H-pyran-2-yl)methyl)-1H-1,2,3-triazol-4-yl)methoxy)benzylidene)hydrazinecarbothioamide (6j)

Brown solid, yield 48.27%, m.p. 125.6–127.3 °C, 99% purity. IR (KBr, *ν*/cm^−1^): 3418, 3268, 1655, 1574, 1445, 1217, 1053, 825, 781. ^1^H NMR (400 MHz, DMSO-*d_6_*) *δ* 7.62 (d, *J* = 11.6 Hz, 1H, –NH–), 7.54 (d, *J* = 4.5 Hz, 1H, ═CH), 7.41 (s, *J* = 11.8 Hz, 1H, ═CH), 7.35 (d, *J* = 4.9 Hz, 1H, ═CH), 7.11 (m, *J* = 5.8 Hz, 1H, Ph-H), 6.72–6.68 (m, 1H, Ph-H), 6.64 (m, *J* = 9.6 Hz, 1H, Ph-H), 6.08 (d, *J* = 9.9 Hz, 1H, ═CH), 5.50–5.44 (m, 2H, –CH_2_), 5.11–5.07 (m, 2H, –CH_2_), 4.30–4.23 (s, 2H, –CH_2_), 2.09 (m, 3H, –CH_3_). ^13^C NMR (101 MHz, DMSO-*d_6_*) *δ* 163.36, 162.72, 148.85, 148.80, 142.12, 141.87, 139.55, 137.27, 135.14, 135.07, 132.42, 132.38, 131.17, 124.18, 123.00, 122.67, 120.93, 120.03, 119.89, 114.44, 112.26, 111.28, 110.74, 73.01, 72.59, 71.57, 71.52, 65.27, 60.42, 60.34, 35.30, 32.24, 32.21, 29.98. HRMS (ESI) *m/z*: [M + H]^+^: 445.1290; found: 445.1289.

#### 2-(3-(Tert-butyl)-2-((1-((5-hydroxy-4-oxo-4H-pyran-2-yl)methyl)-1H-1,2,3-triazol-4-yl)methoxy)benzylidene)hydrazinecarbothioamide (6k)

Yellow solid, yield 34.08%, m.p. 152.6–154.3 °C, 98% purity. IR (KBr, *ν*/cm^−1^): 3420, 3226, 1588, 1525, 1456, 1213, 1056, 797, 762. ^1^H NMR (400 MHz, DMSO-*d_6_*) *δ* 10.08 (s, 1H, –NH), 7.77 (s, 1H, ═CH), 7.73 (s, 1H, ═CH), 7.59 (s, 1H, ═CH), 7.46 (t, *J* = 5.7 Hz, 1H, Ph-H), 7.42 (s, 1H, ═CH), 6.89 (m, *J* = 5.7 Hz, 1H, Ph-H), 6.75–6.66 (m, 1H, Ph-H), 6.14 (s, 1H, ═CH), 5.54 (s, 2H, –CH_2_), 4.94 (s, 2H, –CH_2_), 2.09 (s, 9H, (–C(CH_3_)_3_). ^13^C NMR (101 MHz, DMSO-*d_6_*) *δ* 162.75, 145.88, 134.90, 134.65, 131.42, 123.54, 122.89, 120.83, 120.71, 119.81, 75.76, 71.00, 68.74, 60.60, 48.25, 48.20, 45.06, 45.00, 44.86, 35.37, 31.55. HRMS (ESI) *m/z*: [M + H]^+^: 457.1651; found: 457.1653.

#### 2-(3-((1-((5-Hydroxy-4-oxo-4H-pyran-2-yl)methyl)-1H-1,2,3-triazol-5-yl)methoxy)benzylidene)hydrazinecarbothioamide (6l)

White solid, yield 38.11%, m.p. 172.2–174.3 °C, 97% purity. IR (KBr, *ν*/cm^−1^): 3427, 3124, 1651, 1625, 1578, 1165, 1097, 852. ^1^H NMR (400 MHz, DMSO-*d_6_*) *δ* 11.56 (s, 1H, –NH), 9.38 (s, 1H, ═CH), 8.39 (s, 1H, ═CH), 8.35 (s, 1H, ═CH), 8.23 (s, 1H), 8.08 (d, *J* = 6.5 Hz, 2H, –NH_2_), 7.63 (s, 1H, Ph-H), 7.35 (t, *J* = 7.5 Hz, 1H, Ph-H), 7.30 (d, *J* = 7.1 Hz, 1H, Ph-H), 7.07 (d, *J* = 7.1 Hz, 1H, Ph-H), 6.43 (s, 1H, ═CH), 5.66 (s, 2H, –CH_2_), 5.23 (s, 2H, –CH_2_). ^13^C NMR (101 MHz, DMSO-*d_6_*) *δ* 162.22, 159.38, 148.93, 147.12, 137.29, 134.91, 134.63, 132.50, 128.89, 124.32, 120.86, 117.45, 114.06, 110.95, 109.78, 69.33, 60.46. HRMS (ESI) *m/z*: [M + H]^+^: 401.1027; found: 401.1023.

#### 2-(4-((1-((5-Hydroxy-4-oxo-4H-pyran-2-yl)methyl)-1H-1,2,3-triazol-5-yl)methoxy)benzylidene)hydrazinecarbothioamide (6m)

White solid, yield 39.83%, m.p. 176.7–179.1 °C, 97% purity. IR (KBr, *ν*/cm^−1^): 3478, 3182, 1651, 1613, 1544, 1176, 1131, 858. ^1^H NMR (400 MHz, DMSO-*d_6_*) *δ* 11.33 (s, 1H, –NH), 8.33 (s, 1H, ═CH), 8.04 (s, 1H, ═CH), 7.97 (s, 1H, ═CH), 7.73 (d, *J* = 8.6 Hz, 2H, Ph-H), 7.06 (s, 2H, Ph-H), 6.39 (s, 1H, ═CH), 5.60 (s, 2H, –CH_2_), 5.19 (s, 2H, –CH_2_). ^13^C NMR (101 MHz, DMSO-*d_6_*) *δ* 174.13, 161.03, 159.86, 146.50, 143.41, 142.72, 140.55, 129.41, 127.56, 126.01, 115.37, 113.57, 61.49, 50.47. HRMS (ESI) *m/z*: [M + H]^+^: 401.1027; found: 401.1022.

#### 2-(4-((1-((5-Hydroxy-4-oxo-4H-pyran-2-yl)methyl)-1H-1,2,3-triazol-5-yl)methoxy)-3-methoxybenzylidene)hydrazinecarbothioamide (6n)

White solid, yield 36.89%, m.p. 176.7–178.1 °C, 99% purity. IR (KBr, *ν*/cm^−1^): 3422, 3165, 1658, 1583, 1544, 1142, 1054, 841. ^1^H NMR (400 MHz, DMSO-*d_6_*) *δ* 11.32 (s, 1H, –NH), 9.30 (s, 1H, ═CH), 8.32 (s, 1H, ═CH), 8.16 (s, 1H, ═CH), 8.05 (s, 1H, Ph-H), 7.94 (s, 1H, Ph-H), 7.50 (s, 1H, Ph-H), 7.13 (s, 2H, –NH_2_), 6.40 (s, 1H, ═CH), 5.60 (s, 2H, –CH_2_), 5.15 (s, 2H, –OCH_2_), 3.77 (s, 3H, –CH_3_). ^13^C NMR (101 MHz, DMSO-*d_6_*) *δ* 177.96, 174.15, 161.00, 149.77, 149.59, 146.49, 143.34, 142.98, 140.55, 127.90, 126.17, 122.44, 113.60, 113.35, 109.19, 61.87, 56.10, 50.45. HRMS (ESI) *m/z*: [M + H]^+^: 431.1132; found: 431.1132.

#### 2-(4-((1-((5-Hydroxy-4-oxo-4H-pyran-2-yl)methyl)-1H-1,2,3-triazol-5-yl)methoxy)-3-methylbenzylidene)hydrazinecarbothioamide (6o)

Grey solid, yield 62.03%, m.p. 134.6–136.1 °C, 97% purity. IR (KBr, *ν*/cm^−1^): 3410, 3269, 1646, 1606, 1501, 1214, 1056, 870, 842. ^1^H NMR (400 MHz, DMSO-*d_6_*) *δ* 10.08 (s, 1H, –NH), 8.47 (s, 1H, ═CH), 7.70 (s, 1H, ═CH), 7.56 (s, 1H, ═CH), 7.43 (d, 1H, –NH_2_), 7.17 (s, 1H, Ph-H), 7.05 (d, *J* = 6.1 Hz, 1H, Ph-H), 6.75 (d, *J* = 6.1 Hz, 1H, Ph-H), 6.14 (s, 1H, ═CH), 5.52 (s, 2H, –CH_2_), 5.20 (s, 2H, –CH_2_), 2.73 (s, 3H, –CH_3_). ^13^C NMR (101 MHz, DMSO-*d_6_*) *δ* 170.78, 159.38, 148.93, 146.63, 137.31, 135.08, 134.81, 132.51, 123.73, 122.20, 121.71, 121.68, 120.73, 110.93, 109.85, 69.59, 60.47, 33.25. HRMS (ESI) *m/z*: [M + H]^+^: 415.1179; found: 415.1183.

#### 2-(1-(2-((1-((5-Hydroxy-4-oxo-4H-pyran-2-yl)methyl)-1H-1,2,3-triazol-4-yl)methoxy)phenyl)ethylidene)hydrazinecarbothioamide (6p)

Yellow solid, yield 53.33%, m.p. 124.5–126.4 °C, 97% purity. IR (KBr, *ν*/cm^−1^): 3421, 3208, 1631, 1580, 1569, 1210, 1082, 752. ^1^H NMR (400 MHz, DMSO-*d_6_*) *δ* 7.67 (s, 1H, –NH), 7.55 (s, 1H, –OH), 7.41 (s, 1H, ═CH), 7.12 (s, 1H, ═CH), 6.94 (m, *J* = 6.1 Hz, 1H, Ph-H),6.91–6.88 (m, 1H, Ph-H), 6.81 (d, *J* = 6.6 Hz, 1H, Ph-H), 6.58 (t, *J* = 5.9 Hz, 1H, Ph-H), 6.11 (s, 1H, ═CH), 5.50 (s, 2H, –CH_2_), 5.20 (s, 2H, –CH_2_), 2.75 (s, 3H, –CH_3_). ^13^C NMR (101 MHz, DMSO-*d_6_*) *δ* 163.56, 159.84, 148.65, 145.16, 140.55, 137.94, 134.85, 132.38, 124.60, 124.29, 123.63, 120.84, 120.77, 117.09, 110.89, 110.74, 69.60, 60.45, 39.69, 34.96. HRMS (ESI) *m/z*: [M + H]^+^: 415.1177; found: 415.1183.

#### 2-(1-(4-Fluoro-2-((1-((5-hydroxy-4-oxo-4H-pyran-2-yl)methyl)-1H-1,2,3-triazol-4-yl)methoxy)phenyl)ethylidene)hydrazinecarbothioamide (6q)

Yellow solid, yield 61.5%, m.p. 104.3–105.9 °C, 97% purity. IR (KBr, *ν*/cm^−1^): 3420, 3286, 1649, 1601, 1489, 1213, 1050, 906, 835. ^1^H NMR (400 MHz, DMSO-*d_6_*) *δ* 9.12 (s, 1H, –NH), 7.69 (s, 1H, –OH), 7.55 (s, 1H, ═CH), 7.45 (s, 1H, ═CH), 7.01 (m, *J* = 6.7 Hz, 1H, Ph-H), 6.77 (m, *J* = 9.1 Hz, 1H, Ph-H), 6.44 (m, *J* = 6.7 Hz, 1H, Ph-H), 6.13 (s, 1H, ═CH), 5.51 (s, 2H, –CH_2_), 5.22 (s, 2H, –CH_2_), 2.73 (s, 3H, –CH_3_). ^13^C NMR (101 MHz, DMSO-*d_6_*) *δ* 163.57, 159.37, 152.00, 150.05, 148.87, 146.24, 146.15, 125.45, 125.37, 120.94, 120.72, 110.88, 106.30, 106.13, 101.51, 101.30, 69.87, 60.44, 34.86. HRMS (ESI) *m/z*: [M + H]^+^: 433.1095; found: 433.1089.

#### 2-(1-(4-Chloro-2-((1-((5-hydroxy-4-oxo-4H-pyran-2-yl)methyl)-1H-1,2,3-triazol-4-yl)methoxy)phenyl)ethylidene)hydrazinecarbothioamide (6r)

Brown solid, yield 60.76%, m.p. 187.3–189.5 °C, 97% purity. IR (KBr, *ν*/cm^−1^): 3420, 3256, 1649, 1590, 1483, 1215, 1048, 904, 835. ^1^H NMR (400 MHz, DMSO-*d_6_*) *δ* 9.15 (s, 1H, –NH), 8.45 (s, 1H, –OH), 7.68 (s, 1H, ═CH), 7.45 (s, 1H, ═CH), 7.00–6.97 (m, 1H, Ph-H), 6.91 (d, *J* = 6.7 Hz, 1H, Ph-H), 6.62 (m, *J* = 6.6 Hz, 1H, Ph-H), 6.13 (s, 1H, ═CH), 5.51 (s, 2H, –CH_2_), 5.23 (s, 2H, –CH_2_), 2.73 (s, 3H, –CH_3_). ^13^C NMR (101 MHz, DMSO-*d_6_*) *δ* 163.61, 159.36, 148.88, 145.75, 139.51, 137.30, 134.50, 132.49, 127.92, 125.33, 122.70, 120.91, 117.02, 111.23, 110.88, 69.91, 60.34, 34.78. HRMS (ESI) *m/z*: [M + H]^+^: 449.0797; found: 449.0793.

#### 2-(1-(4-Bromo-2-((1-((5-hydroxy-4-oxo-4H-pyran-2-yl)methyl)-1H-1,2,3-triazol-4-yl)methoxy)phenyl)ethylidene)hydrazinecarbothioamide (6s)

Yellow solid, yield 47.45%, m.p. 112.0–113.1 °C, 97% purity. IR (KBr, *ν*/cm^−1^): 3425, 3295, 1652, 1583, 1461, 1216, 1067, 887, 832. ^1^H NMR (400 MHz, DMSO-*d_6_*) *δ* 9.15 (s, 1H, –NH), 8.45 (s, 1H, –OH), 7.67 (s, 1H, ═CH), 7.45 (s, 1H, ═CH), 7.00 (m, *J* = 6.5 Hz, 1H, Ph-H), 6.93 (d, *J* = 6.5 Hz, 1H, Ph-H), 6.75–6.71 (m, 1H, Ph-H), 6.13 (s, 1H, ═CH), 5.51 (s, 2H, –CH_2_), 5.23 (s, 2H, –CH_2_), 2.72 (s, 3H, –CH_3_). ^13^C NMR (101 MHz, DMSO-*d_6_*) *δ* 163.84, 163.61, 159.37, 148.87, 145.77, 139.55, 137.32, 134.51, 132.48, 125.54, 123.00, 120.90, 119.49, 118.69, 113.46, 110.87, 69.92, 60.44, 34.75. HRMS (ESI) *m/z*: [M + H]^+^: 493.0293; found: 493.0288.

#### 2-(1-(2-((1-((5-Hydroxy-4-oxo-4H-pyran-2-yl)methyl)-1H-1,2,3-triazol-4-yl)methoxy)-4-methoxyphenyl)ethylidene)hydrazinecarbothioamide (6t)

White solid, yield 68.94%, m.p. 181.6–183.5 °C, 99% purity. IR (KBr, *ν*/cm^−1^): 3458, 3253, 1647, 1605, 1497, 1219, 1092, 856, 810. ^1^H NMR (400 MHz, DMSO-*d_6_*) *δ* 9.05 (s, 1H, –NH), 8.45 (s, 1H, –OH), 7.67 (s, 1H, ═CH), 7.45 (s, 1H, ═CH), 6.92 (d, *J* = 6.8 Hz, 1H, Ph-H), 6.46 (d, *J* = 6.8 Hz, 1H, Ph-H), 6.23 (m, *J* = 6.9 Hz, 1H, Ph-H), 6.12 (s, 1H, ═CH), 5.51 (s, 2H, –CH_2_), 5.20 (s, 2H, –CH_2_), 4.03 (s, 3H, –CH_3_), 2.73 (s, 3H, –CH_3_). ^13^C NMR (101 MHz, DMSO-*d_6_*) *δ* 163.37, 159.36, 149.38, 148.92, 146.11, 140.52, 137.29, 134.80, 132.49, 124.99, 120.79, 117.67, 110.85, 105.02, 100.38, 69.64, 64.78, 60.43, 34.94. HRMS (ESI) *m/z*: [M + H]^+^: 445.1290; found: 445.1289.

#### 2-(1-(2-((1-((5-Hydroxy-4-oxo-4H-pyran-2-yl)methyl)-1H-1,2,3-triazol-4-yl)methoxy)-4-methylphenyl)ethylidene)hydrazinecarbothioamide (6u)

Brown solid, yield 65.47%, m.p. 93.9–96.1 °C, 97% purity. IR (KBr, *ν*/cm^−1^): 3420, 3247, 1650, 1608, 1489, 1218, 1088, 907, 813. ^1^H NMR (400 MHz, DMSO-*d_6_*) *δ* 8.32 (s, 1H, –NH), 8.16 (s, 1H, –OH), 8.02 (s, 1H, ═CH), 7.60 (s, 1H, ═CH), 7.53 (m, *J* = 7.9 Hz, 1H, Ph-H), 7.34 (m, *J* = 7.7 Hz, 1H, Ph-H), 6.78 (m, *J* = 7.8 Hz, 1H, Ph-H), 6.37 (s, 1H, ═CH), 5.62 (s, 2H, –CH_2_), 5.21 (s, 2H, –CH_2_), 2.32 (s, 3H, –CH_3_), 2.16 (s, 3H, –CH_3_). ^13^C NMR (101 MHz, DMSO-*d_6_*) *δ* 178.84, 163.47, 145.12, 140.62, 136.12, 134.89, 132.57, 132.36, 124.24, 124.11, 121.35, 120.79, 120.72, 117.57, 111.43, 69.85, 69.56, 60.44, 45.84, 37.54, 37.39, 34.93. HRMS (ESI) *m/z*: [M + H]^+^: 429.1344; found: 429.1340.

#### 2-(1-(3-((1-((5-Hydroxy-4-oxo-4H-pyran-2-yl)methyl)-1H-1,2,3-triazol-4-yl)methoxy)phenyl)ethylidene)hydrazinecarbothioamide (6v)

White solid, yield 59.86%, m.p. 204.3–207.3 °C, 98% purity. IR (KBr, *ν*/cm^−1^): 3430, 3302, 1624, 1578, 1467, 1202, 1073, 820, 723. ^1^H NMR (400 MHz, DMSO-*d_6_*) *δ* 9.31 (s, 1H, –NH), 8.34 (s, 1H, ═CH), 8.06 (s, 1H, ═CH), 7.61–7.59 (m, 1H, Ph-H), 7.47 (d, *J* = 7.8 Hz, 1H, Ph-H), 7.30 (t, *J* = 8.0 Hz, 1H, Ph-H), 7.06 (m, *J* = 7.9 Hz, 1H, Ph-H), 6.40 (s, 1H, ═CH), 5.62 (s, 2H, –CH_2_), 5.23 (s, 2H, –CH_2_), 2.29 (s, 3H, –CH_3_). ^13^C NMR (101 MHz, DMSO-*d_6_*) *δ* 159.35, 148.93, 146.85, 138.61, 137.30, 135.02, 132.49, 131.78, 127.98, 123.90, 120.82, 116.11, 113.10, 110.90, 110.38, 69.25, 60.43, 31.80. HRMS (ESI) *m/z*: [M + H]^+^: 415.1181; found: 415.1183.

#### 2-(1-(4-((1-((5-Hydroxy-4-oxo-4H-pyran-2-yl)methyl)-1H-1,2,3-triazol-5-yl)methoxy)phenyl)ethylidene)hydrazinecarbothioamide (6w)

White solid, yield 74.86%, m.p. 128.6–132.5 °C, 99% purity. IR (KBr, *ν*/cm^−1^): 3410, 3205, 1627, 1604, 1468, 1221, 1095, 837. ^1^H NMR (400 MHz, DMSO-*d_6_*) δ 9.10 (s, 1H, –NH), 8.45 (s, 1H, –OH), 7.68 (s, 1H, ═CH), 7.45 (s, 1H, ═CH), 7.32 (d, *J* = 7.1 Hz, 1H, Ph-H), 6.63 (d, *J* = 7.2 Hz, 1H, Ph-H), 6.12 (s, 1H, ═CH), 5.50 (s, 2H, –CH_2_), 5.17 (s, 2H, –CH_2_), 2.81 (s, 3H, –CH_3_). ^13^C NMR (101 MHz, DMSO-*d_6_*) δ 174.11, 161.00, 160.58, 157.02. HRMS (ESI) *m/z*: [M + H]^+^: 415.1184; found: 415.1183.

#### 2-(1-(4-((1-((5-Hydroxy-4-oxo-4H-pyran-2-yl)methyl)-1H-1,2,3-triazol-5-yl)methoxy)-3-methylphenyl)ethylidene)hydrazinecarbothioamide (6x)

Yellow solid, yield 39.67%, m.p. 187.1–190.4 °C, 99% purity. IR (KBr, *ν*/cm^−1^): 3415, 3248, 1641, 1619, 1575, 1222, 1025, 876, 804. ^1^H NMR (400 MHz, DMSO-*d_6_*) *δ* 7.68 (d, *J* = 11.6 Hz, 1H, –NH), 7.59 (s, 1H, –OH), 7.41 (s, 1H, ═CH), 7.32 (d, 1H, ═CH), 7.26 (d, *J* = 1.4 Hz, 1H, Ph-H), 7.14 (m, *J* = 6.9 Hz, 1H, Ph-H), 6.69 (d, *J* = 7.0 Hz, 1H, Ph-H), 6.10 (s, 1H, ═CH), 5.49 (m, 2H, –CH_2_), 5.18 (m, 2H, –CH_2_), 2.80 (s, 3H, –CH_3_), 2.73 (m, 3H, –CH_3_). ^13^C NMR (101 MHz, DMSO-*d_6_*) *δ* 163.29, 159.88, 148.67, 146.19, 138.80, 135.14, 132.38, 124.43, 123.46, 121.16, 121.05, 120.67, 110.80, 109.47, 69.55, 60.46, 59.33, 33.29, 31.51. HRMS (ESI) *m/z*: [M + H]^+^: 429.1340; found: 429.1340.

### *In vitro* tyrosinase inhibition

The mushroom tyrosinase inhibitory activity was determined according to previous described method^32^. Briefly, 168 μl of phosphate buffer (0.1 M, pH 6.8), 10 μl of mushroom tyrosinase (0.5 mg/ml, Sigma Chemical, St. Louis, MO) and 2 μl of the inhibitor solution were placed in the wells of a 96-well micro plate. After pre-incubation for 20 min at 37 °C, 20 μl of 2.0 mg/ml l-DOPA (3,4-dihydroxyphenylalanine, Sigma Chemical, St. Louis, MO) was added and the enzyme activity was measured at 475 nm every 60 s for 180 s in a Microplate Reader (Bio-Rad Laboratories, Inc., Hercules, CA). Kojic acid and phosphate buffer were respectively used as positive and negative control. The extent of inhibition by the test compounds was expressed as the percentage of concentration necessary to achieve 50% inhibition (IC_50_). The percentage of inhibition was calculated as follows: inhibitory rate (%) = [*A*_c_ − *A*_t_)/*A*_c_] × 100. *A*_c_ is the absorbance of the negative control and *A*_t_ is the absorbance of the test compound. Each concentration was analysed in three independent experiments run in triplicate. The IC_50_ values were determined by the data analysis software GraphPad Prism 8 (La Jolla, CA).

### Tyrosinase kinetic analysis

To study the inhibition type of kojic acid-triazole derivatives, the investigation of inhibitory type of selected compound **6w** on mushroom tyrosinase was carried out according to reported protocol^33^. Compound **6w** was used at the concentrations of 0 μM, 2 μM, and 4 μM, respectively. Substrate l-DOPA concentration was between 500 µM and 1000 µM in the process of all kinetic study. Pre-incubation time and measurement time were the same as described in mushroom tyrosinase inhibition assay procedure. Lineweaver–Burk’s plots of inverse of velocities (1/*V*) versus inverse of substrate concentration 1/[*S*] µM^−1^ were used to determine the type of enzyme inhibition.

### Measurements of fluorescence spectra

To investigate fluorescence quenching role of inhibitor to the tyrosinase, fluorescence experiments were carried according to the reported method^34^. To determine the linear concentration range of the fluorescence, compound **6w** was first dissolved in DMSO. The concentration of the solution was 10 mM, and then was diluted with sodium phosphate buffer. The ultimate concentration was changed from 0 to 50 μM. A series of 3 ml solutions containing 1.5 ml of tyrosinase solution were added to a centrifuge tube and then accurately mixed with different concentrations of **6w** solution range from 0 to 50 μM. Cary Eclipse G9800A fluorescence spectrophotometer (Agilent Technologies Co., Santa Clara, CA) was employed to detect the fluorescence intensity. The excitation wavelength was set to 280 nm, the bath was set as three temperatures (292 K, 298 K, and 304 K) with a scanning wavelength change from 300 to 500 nm. The excitation and emission bandwidths were 5 nm. The final tyrosinase concentration was 0.05 mg/ml.

### FT-IR spectrum measurements

A Nicolet-iS5 spectrophotometer was used to collect FT-IR spectra with germanium attenuated total reflection (ATR) based on the reported method^35^. 0.5 mg/ml tyrosinase in PBS buffer solution, pH = 6.8 PBS buffer solution and 10 µM compound **6w** in DMSO was used in the experiment. Tyrosinase solution and complex solution were smeared on KBr tablets for spectral measurement, respectively. The spectra of tyrosinase and compound **6w**–tyrosinase complex were measured with the wavenumber of 4000–500 cm^−1^, a resolution of 4 cm^−1^, and 64 scans. The blank PBS buffer solution was measured as the baseline. The compositions of tyrosinase’s secondary structures were analysed using PeakFit software.

### UV–vis experiment

UV-2600 spectrophotometer (Shimadzu Corporation, Tokyo, Japan) is used to determine UV–vis absorption spectra. PBS solution with pH 6.8 was adopted as a reference. At 298 K, UV–vis absorption spectra of tyrosinase, compound **6w** along with the compound **6w**–tyrosinase complex were scanned with gap (1 nm) and wavelength range (200–600 nm). The blank PBS buffer solution was measured as the baseline. Ten micromolar of the compound **6w** and tyrosinase were set for UV–vis analysis^36^.

### 3D fluorescence spectra

3D fluorescence spectra were conducted as previous reports with slit width of 2.5 nm and scan rate of 1200 nm/min using fluorescence spectrometer (FL-970, Techcomp, Shanghai, China)^37^. The solution of compound **6w** in DMSO was added into tyrosinase solution and incubated for 10 min. 3D fluorescence spectra were detected at 250–900 nm. The concentration of tyrosinase and compound **6w** was 0.5 mg/ml μM and 10 μM, respectively.

### *In silico* docking simulation of tyrosinase with compound 6w

Performing molecular docking calculations by using the X-ray structure of mushroom tyrosinase in complex with tyrosinase inhibitor tropolone (PDB ID: 2Y9X). The most potent compound **6w** was prepared by conducting prepare ligands procedure (Discovery studio 2019) in their neutral form and preferential conformation was obtained in the CHARMm force field. Choosing one of the eight monomers from the PDB entry, and the protein structure was prepared through performing the Prepara Protein module via using Discovery studio 2019 software, the specific steps including adding missing residues and hydrogen atoms as well as removing water molecules and spectator ions. Then, their conformation optimised in the CHARMm force field. The search grid of binding site was identified as centre_*x*: −10.021, centre_*y*: −28.823, and centre_*z*: −43.596 with radius value of 10. Molecular modelling simulations were performed with CDOCKER protocol (Discovery studio 2019), reporting the top 10 poses for each ligand and the graph generation was done by PyMOL (Schrödinger, New York, NY)^33^^,^^38^.

### Effects on the browning process of fresh-cut apples assay

Fuji apples were planted in Yantai, China, and were bought from Walmart supermarket in Shaoyang, China. The apples were come from the same batch having similar shapes, maturity, and without injuries, rot, diseases, or pests. Apples were cut into thin pieces with same size by using a sharp knife. Then, the fresh-cut slices were divided randomly into different groups: blank control group was immersed in ethanol for 10 min at room temperature, treatment groups were dipped in the solution of compound **6w** under the same conditions, positive groups were dipped in 50 μM kojic acid and 50 μM vitamin C under the same conditions. The test samples were air-dried naturally and immediately were separately packaged with polyethylene clam-packs, and stored at 2–8 °C, and used for the following experimental measurements.

According to the reported protocol^39^, the colour of apple samples was tested by a CR-400 Minolta chronometer instrument (Konica Minolta, Osaka, Japan). Three data of L*, a*, and b* were recorded for each sample. The L* values indicate lightness, the a* values show reddish-greenish, the b* values represent yellowish-bluish. The colour measurement was carried out in triplicate by random sampling.

### DPPH (2,2-diphenyl-1-picrylhydrazyl) free radical scavenging activity assay

The anti-oxidation effect on DPPH radical was determined by a slightly modified version of a previously described DPPH radical scavenging assay^40^. Reaction mixtures consisted of 100 μl of DPPH (150 μM), 20 μl of increasing concentration of test compounds and the volume was adjusted to 200 μl in each well with DMSO. The assay solution was then incubated at room temperature (25 °C) for 30 min in the dark. After the incubation, absorbance at 517 nm was measured by using UV-2600 spectrophotometer. The experiments were undertaken in triplicate. DMSO was used as a control. Ascorbic acid was used as antioxidant standard for comparison of the activity. The calculation of DPPH radical scavenging activity was followed by this formula:

$$\begin{array}{l} \text{DPPH radical scavenging activity }\left(\%\right)\\ =\frac{\left(\text{absorbance of standard}-\text{absorbance of sample}\right)}{\text{Absorbance of standard}}\times 100 \end{array}$$

### ABTS (2,2′-azino-bis-(3-ethylbenzthiazoline-6-sulfonic acid)) radical cation scavenging activity assay

ABTS free radical scavenging activity was performed according to the procedure described by Zhang et al.^40^ Test solution of ABTS decolourisation assay was prepared by mixing 2.45 mM potassium persulfate and 7 mM ABTS at 8:12 volume/volume ratio. The working solution was kept in the dark at room temperature for 12–18 h. Before ABTS radical scavenging test, the ABTS test solution was diluted with DMSO to absorbance of 0.7 (±0.02) at 734 nm. One millilitre of diluted ABTS test solution was added to 10 μl of DMSO or test sample. After initial mixing, the working solution was kept in the dark at room temperature for 2–6 min, and the absorbance was measured at 734 nm. This test was carried out in triplicate. DMSO was used as a control. Ascorbic acid was used as antioxidant standard for comparison of the activity. The absorbance of test samples was calculated by the following formula.

$$\begin{array}{l} \text{ABTS scavenging ability }\left(\%\right)\\ =\frac{\left(\text{absorbance of standard}-\text{absorbance of sample}\right)}{\text{absorbance of standard}}\times 100. \end{array}$$

## Results and discussion

### Chemistry

The synthesis route of kojic acid triazol thiosemicarbazide Schiff base derivatives is shown in Scheme 1. Propargyl ether compounds were synthesised from different substituted hydroxyl aromatic aldehydes or ketones and propargyl bromide, and the triazol thiosemicarbazide Schiff base derivatives can be obtained by the condensation reaction of thiosemicarbazide with different propargyl ether in ethanol. 2-(Azidomethyl)-5-hydroxy-4H-pyran-4-one was synthesised from kojic acid and sodium azide. Kojic acid triazol thiosemicarbazide Schiff base derivatives were obtained by the cyclisation reaction. In this present study, 24 kojic acid triazol thiosemicarbazide Schiff base derivatives were synthesised. The yields of the synthesis products were not high. All the target compounds can be dissolved in alcohol, ethyl acetate, DMF, DMSO, etc. The synthesised compounds have been characterised by ^1^H NMR, ^13^C NMR, IR, and HRMS. IR spectra of all target compounds illustrated stretching band associated with (C═N) at about 1590 cm^−1^. In ^1^H NMR spectrum, ═CH proton appeared as singlet at about 9.30–10.08 ppm. Signals in the range of 6.10–8.30 ppm region belong to aromatic, kojic acid, or thiazol protons. In ^13^C NMR spectrum, the signal appeared at about 170 ppm was assigned to the C═N.

### Tyrosinase inhibitory activity

**Figure 1.**
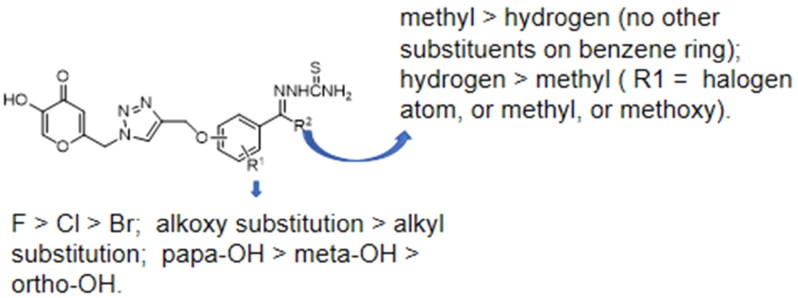
Structure–activity relationship of kojic acid triazol thiosemicarbazide Schiff base derivatives.

The tyrosinase inhibition measurements of compounds **6a–6x** were carried out using l-DOPA as substrate. Kojic acid was selected as a positive control compound. The inhibitory activities of the kojic acid triazol thiosemicarbazide Schiff base derivatives against tyrosinase are presented in Table 1. All the synthesis compounds displayed potent inhibitory activities against mushroom tyrosinase with IC_50_ values ranged from 0.94 ± 0.035 μM to 22.89 ± 0.057 μM. From Table 1, except for compound **6h**, the other compounds showed better inhibitory effects on tyrosinase than the positive control inhibitor kojic acid, and compound **6w** was the most potent tyrosinase inhibitor with IC_50_ value of 0.94 μM ± 0.035. R^2^ is hydrogen (Figure 1): (1) R^1^ is 4-halogen substitution (**6b–6d**), fluorine substitution compound expressed more potent inhibitory activity than the other compounds and positive control inhibitor kojic acid, and the inhibitory activity gradually decreases with the increase halogen atom radius. The same rule can be observed when R^1^ is 5-halogen substitution (**6g–6h**); (2) the inhibitory activity of compound with alkoxy substitution on benzene ring is slightly improved, and the inhibitory activity of alkoxy substituted compounds is better than that of alkyl substituted compounds. The results indicate that the presence of electron-donating groups might have important influence to the inhibitory activity; (3) comparing the inhibitory activity of compounds **6a**, **6l**, **6m**, compound with hydroxyl group at papa position had the best inhibitory activity. Moreover, inhibitory activity of compound with hydroxyl group at papa position is better than the inhibitory activity of compound with hydroxyl group at meta position, and inhibitory activity of compound with hydroxyl group at meta position is better than the inhibitory activity of compound with hydroxyl group at ortho position. The results showed that inhibitory activity may be related to steric hindrance. R^2^ is methyl (Figure 1): (1) there are no other substituents on benzene ring, the same law can be obtained as R^2^ is hydrogen, and the inhibitory activity of compound with methyl (R^2^) is better than the compound with hydrogen (R^2^); (2) when R^1^ is substituted by halogen atoms, it can be found that the inhibitory activity of fluorine substitution is stronger than that of chlorine substitution and bromine substitution. In addition, with the increase of atomic radius, the inhibitory activity gradually decreases. (3) The inhibitory activity of alkoxy substituted compound is better than that of alkyl substituted compound. (4) Comparing the tyrosinase inhibitory activity of compounds with halogen atom (**6b–6d**, **6q–6s**) and compounds with methyl or methoxy (**6e–6f**, **6t–6u**), when the hydrogen (R^2^) was replaced by the methyl (R^2^), the inhibitory activity was decreased.

**Table 1.** Inhibitory tyrosinase activity of the target compounds and kojic acid.

Compd	IC_50_ (μM) ± SD	Compd	IC_50_(μM) ± SD
**6a**	10.17 ± 0.021	**6n**	8.40 ± 0.009
**6b**	7.06 ± 0.038	**6o**	12.38 ± 0.014
**6c**	8.29 ± 0.062	**6p**	14.59 ± 0.014
**6d**	12.34 ± 0.015	**6q**	9.58 ± 0.037
**6e**	9.04 ± 0.021	**6r**	11.51 ± 0.030
**6f**	10.78 ± 0.066	**6s**	14.91 ± 0.025
**6g**	9.34 ± 0.057	**6t**	12.75 ± 0.011
**6h**	22.89 ± 0.057	**6u**	15.76 ± 0.041
**6i**	18.24 ± 0.043	**6v**	1.20 ± 0.015
**6j**	8.49 ± 0.028	**6w**	0.94 ± 0.035
**6k**	10.25 ± 0.031	**6x**	2.35 ± 0.021
**6l**	5.45 ± 0.077	Kojic acid	22.04 ± 0.033
**6m**	1.58 ± 0.092		

### Inhibition mechanism

**Figure 2.**
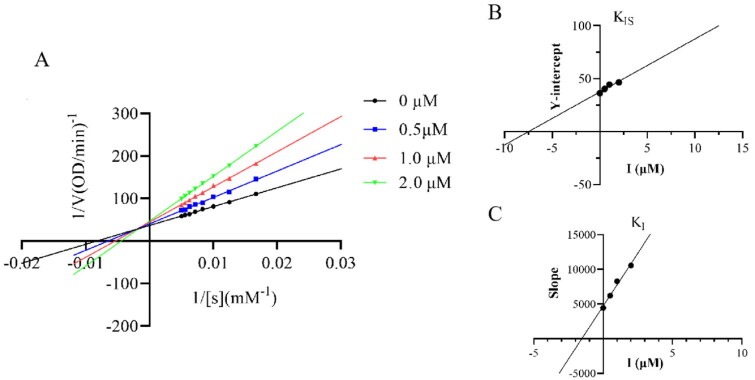
Kinetic inhibition measurement of compound **6w**. The type of inhibition was exhibited by the Lineweaver–Burk plot. (A) The concentrations of compound **6w** in the figure were 0, 0.5 μM, 1 μM, and 2 μM, respectively. (B) The secondary replot indicates the intercept versus the concentrations of compound **6w** to define the inhibition constant Kis. (C) The secondary replot indicates the slope versus the concentrations of compound **6w** to define the inhibition constant Ki.

Among the assessed kojic acid triazol thiosemicarbazide Schiff base derivatives, compound **6w** expressed better tyrosinase inhibitory activity than the other compounds, in order to further explore the inhibition mechanism, the inhibitory type of the compound **6w** on mushroom tyrosinase was decided by the Lineweaver–Burk double reciprocal plots. The Lineweaver–Burk plots were obtained by different concentrations of the compound **6w** and the substrate (Figure 2(A)). The inhibition type of the compound **6w** was studied as shown in Figure 2(A). The plots of 1/*V* versus 1/[*S*] gave a family of straight lines with different slopes. The slope of the curve showed downtrend with the decrease of the inhibitor concentration. All of the straight lines intersected at a point in the second quadrant. The results indicated that compound **6w** was mixed type inhibitor. The inhibition constant (*Ki*) of the compound was obtained by a plot of the slope versus the concentrations of the compound **6w**, *Kis* of the compound was obtained by a plot of vertical intercept (1/*V_m_*) versus the concentrations of the compound **6w**, the results were shown in Figure 2(B,C). The *Ki* value and the *Kis* value of the compound were 0.0003 µM and 0.1997 µM, respectively. Compound **6w** has a better binding with free enzyme.

### Fluorescence quenching

**Figure 3.**
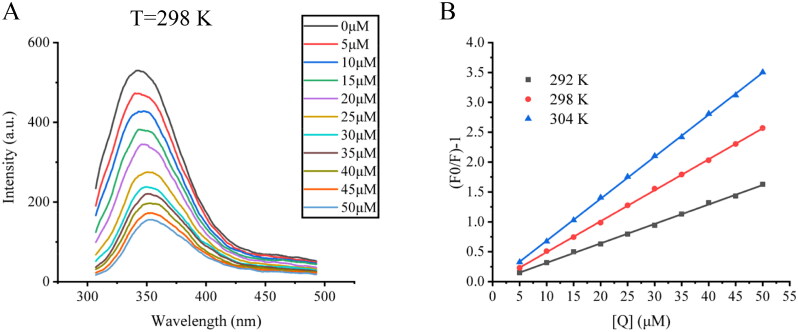
(A) Changes in the tyrosinase fluorescence at different concentrations of compound **6w** (*λ*_ex_ = 280 nm, *T* = 298 K). The final enzyme concentration was 0.05 mg/ml, and c (compound **6w**) = 0 μM, 5 μM, 15 μM, 20 μM, 25 μM, 30 μM, 35 μM, 40 μM, 45 μM, and 50 μM for the curves from top to bottom, respectively. (B) The Stern–Volmer figure of compound **6w** and tyrosinase at 292, 298, and 304 K.

Fluorescence quenching means that some small molecule compounds bind to protein that can affect the fluorescence intensity of the fluorophore. Therefore, in order to obtain information about conformational changes of enzymes, fluorescence spectroscopy has become a very important research tool. Fluorescence spectra of tyrosinase with different concentrations of compound **6w** were investigated, as shown in Figure 3. With increasing concentrations of compound **6w**, the dramatically decreasing fluorescence intensity of tyrosinase could be found. This suggested the inhibitor had potent quenching effect on the fluorescence of tyrosinase. At the same time, there was no significant red shift or blue shift in the fluorescence emission peak of tyrosinase after the addition of compound **6w**. This result showed that the interaction between the sample and tyrosinase did not change the hydrophobic environment in the vicinity of the chromophore tryptophan (Trp) residues. The Stern–Volmer figure of compound **6w** with tyrosinase at 292, 298, and 304 K is shown in Figure 3(B). Stern–Volmer’s plots showed a good linear relationship, it suggested that the quenching type for compound **6w** was single. Besides, it also suggested that the dominant quenching mechanism of compound **6w** should be a dynamic quenching for the quenching constant increased with enhanced temperature. The quenching of the intrinsic fluorescence provided a direct and clear evidence that the inhibitor **6w** was capable of binding to tyrosinase, and the binding of **6w** to tyrosinase led to change in the microenvironment around the fluorophore, and the detailed *K*_SV_ values are summarised Table 2.

**Table 2.** Stern–Volmer equation parameters for interaction between **6w** and tyrosinase.

*T* (K)	R^2^	*K*_SV_ (L/µmol)	Kq (L/µmol/s)
292 K	0.99862	0.030052	3.0052 × 10^7^
298 K	0.99959	0.046048	4.6048 × 10^7^
304 K	0.99973	0.065292	6.5292 × 10^7^

### FT-IR spectrum

**Figure 4.**
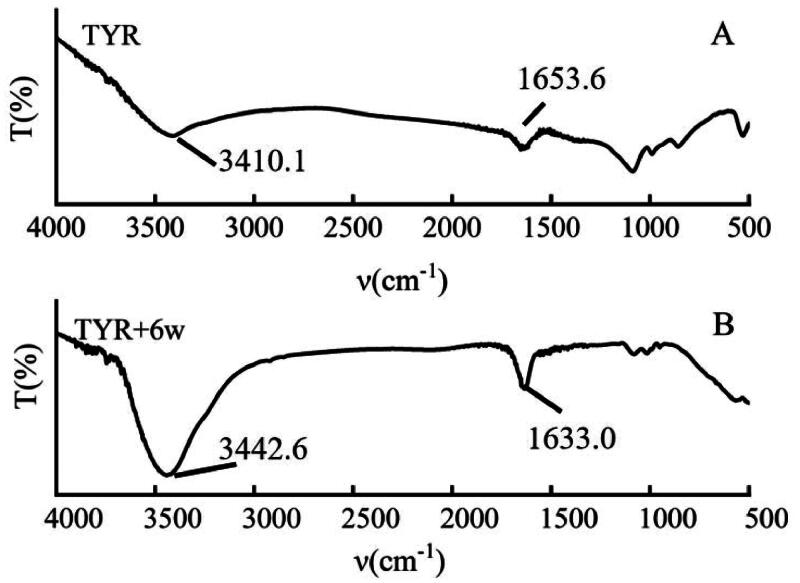
FT-IR spectra of tyrosinase with compound **6w**.

FT-IR spectroscopy has been widely used to study the secondary structure conformation of small molecules and proteins systems. Amide I band (1600–1800 cm^−1^) is ascribed to the stretching vibration of C═O group, and amide II band (3300–3500 cm^−1^) corresponds to the bending vibration of N–H^36^. The interaction between compound **6w** and tyrosinase was studied by FT-IR spectroscopy (Figure 4). The spectral results indicated that the characteristic peak of the amide I band shifted from 1653 to 1633 cm^−^1 and the characteristic peak of the amide II band shifted from 3410 to 3442 cm^−^1. The results showed that the compound **6w** interacted with the C═O and N–H moieties of polypeptides and caused a rearrangement in the folding conformation, resulting in potential inhibitory activity against tyrosinase.

### UV–vis spectrum

**Figure 5.**
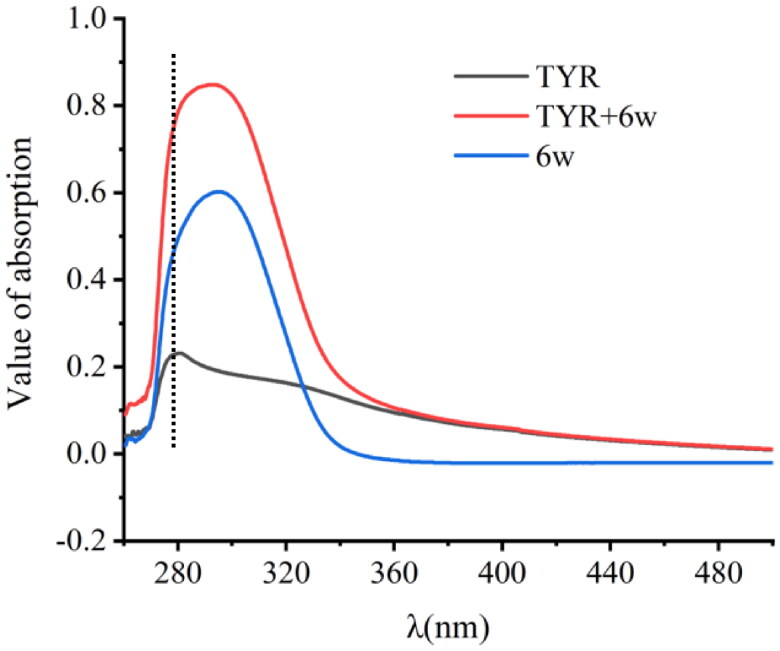
UV–vis spectra of tyrosinase with compound **6w**.

UV–vis absorption spectroscopy can be used to evaluate the conformational change of protein structure and interaction between small molecule and protein^41^. The peak near 280 nm is mainly due to the n→π* transition, reflecting information of the microenvironment changes around Trp and Tyr residues^42^. After adding the compound **6w**, the absorbance of tyrosinase increased, while the absorption peak did not move significantly (Figure 5). The changes of characteristic peak indicated that compound **6w** bound with tyrosinase, resulting in conformational changes in its secondary structure of tyrosinase and the formation of compound **6w**–tyrosinase complex. In addition, in terms of fluorescence emission spectra, it has been proved that dynamic quenching only impacts its excited state and does not cause the changes in absorption spectrum. Therefore, the result of UV–vis spectral was consistent with the result of subsequent fluorescence spectrum.

### 3D fluorescence spectra

**Figure 6.**
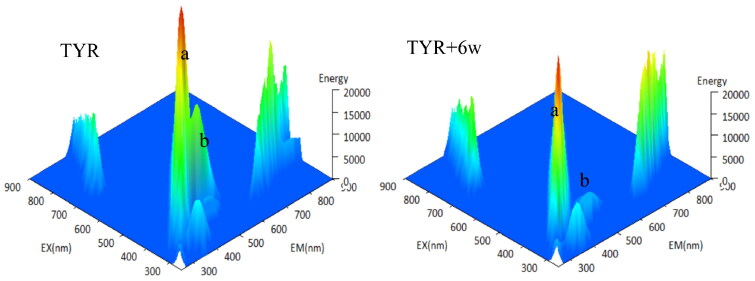
3D fluorescence spectra of tyrosinase (A) and tyrosinase-paeonol (B).

3D fluorescence spectra are performed to further explore the effects of compound **6w** on the conformational and microenvironment change of tyrosinase and the results are shown in Figure 6. Combining previous literature results^43^, tyrosinase showed two characteristic peaks in its 3D fluorescence spectra, peak a (*λ*_ex_/*λ*_em_ = 278/338 nm, Tyr and Trp residue) and peak b (*λ*_ex_/*λ*_em_ = 232/335 nm, polypeptide strand transition), respectively (Figure 6(A)). Interesting, compound **6w** treatment decreased the intensity of peak b (Figure 6(B)) obviously, suggesting that the interaction between compound **6w** and tyrosinase caused the perturbation of the tyrosinase polypeptide strand and subsequently conformational transition.

### Molecular docking study

**Figure 7.**
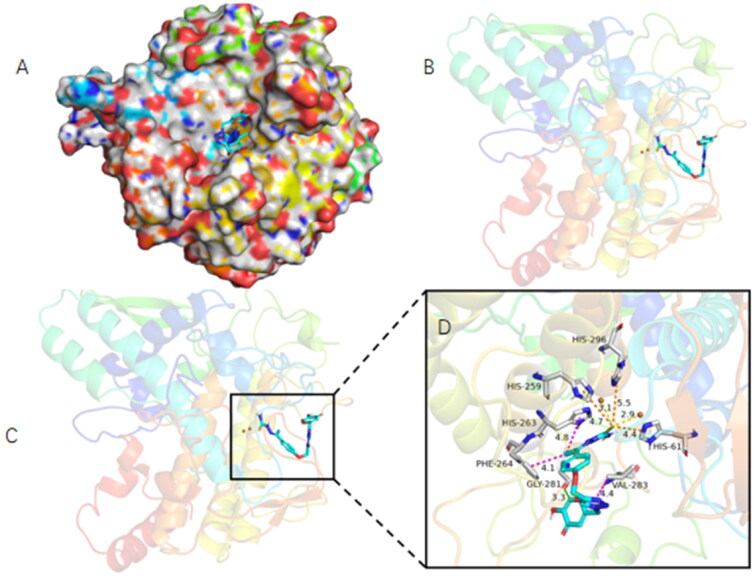
(A) Docking model of the best binding pose for compound **6w** to tyrosinase. (B) The simulated docking model of the interactions of compound **6w** with active site of mushroom tyrosinase by AutoDock4.2. (C) Superposition of **6w**-bound with mushroom tyrosinase. (D) The schematic representation of the interactions of compound **6w** in the binding pocket of mushroom tyrosinase derived from the docking model. Dashed lines represent bond distances between interacting functionalities of the ligand and receptor. The legend inset represents the type of interaction between the ligand atoms and the amino acid residues of the protein.

To clarify whether there is a direct binding of **6w** to the tyrosinase, we further performed the molecular docking study by using Discovery Studio 2019 software (Figure 7(A–D)). As shown in Figure 7(A), **6w** has been identified as a mixed type inhibitor through the kinetic assay. In Figure 7, the results showed that thiosemicarbazide fragment in **6w** entered the bottom of the tyrosinase activity pocket, which not only well overlapped with the original ligand of the crystal structure but also well overlapped with thiosemicarbazide fragment. The sulphur atom of the thiosemicarbazide fragment interacted with copper ions through a coordination bond, and the bond lengths are 2.9 Å and 3.1 Å, respectively. The pi-sulphur bone presented between thiosemicarbazide fragment and His 61, His 259, and His 296 with the bond lengths 4.4 Å, 4.7 Å, and 5.5 Å, respectively. The above results further confirmed that compound **6w** was a mixed type inhibitor. The kojic acid fragment formed a significant hydrogen bond interaction with Gly 281 with a bond length of 3.3 Å. Triazole ring interacted with Val 283 by pi-alkyl bone, and the bond length is 4.4 Å. The pi-alkyl bone also presented between methyl group and His 263 and Phe 264 with the bond lengths 4.8 Å and 4.1 Å, respectively. The docking simulation results indicated that the best inhibitor **6w** could bind to the mushroom tyrosinase active site and directly suppress tyrosinase activity. Kojic acid is an important fragment to improve the inhibitory activity.

### Anti-browning study

**Figure 8.**
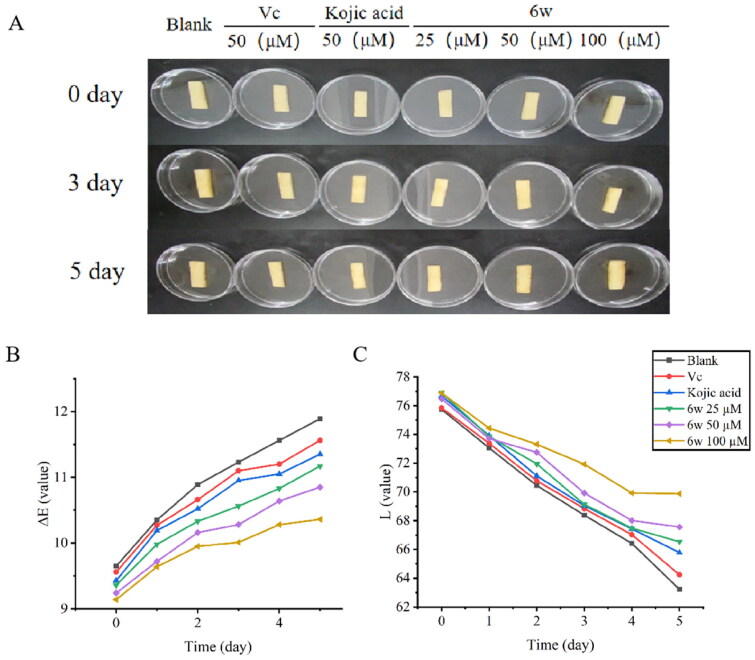
(A) Photographs of fresh-cut apples before treatment, control fresh-cut apples after five days of storage at 5 °C and **6w**-treated (25 μM, 50 μM, and 100 μM) fresh-cut apples after five days of storage at 5 °C. (B, C) Effects of compound **6w** in controlling the browning of fresh-cut apple during the 5 day-storage at 5 °C.

Among the synthesised target compounds, the candidate **6w** showed the best tyrosinase inhibitory activity. Therefore, we further evaluated its anti-browning effect on apple, in which kojic acid and vitamin C were used as positive control. The colour value change of post-cut apple treated with **6w** (25 μM, 50 μM, and 100 μM), blank control group, kojic acid (50 μM), and vitamin C (50 μM) during storage at 5 °C is shown in Figure 8(A). The L* values of the **6w** (25 μM) group, **6w** (50 μM) group, **6w** (100 μM) group, kojic acid group, vitamin C group, and blank group were gradually decreased, after storage at 5 °C for five days. The L* values of the different concentration compound **6w** groups were not only significantly larger than blank control group but also slight larger than both vitamin C and kojic acid. During the measurement (0–5 days), the browning extent of the apple slices in the treatment groups was obviously lower than the browning extent of the apple slices in the blank control group, kojic acid group, and vitamin C group. Furthermore, the colour of the apple slices in the compound **6w** group is lighter than the colour of the positive group after storage at 5 °C for five days (Figure 8(B)). The results further suggested that compound **6w** could be used as a potent anti-browning agent, and the anti-browning ability became stronger with the increase of concentration.

### Antioxidant activities

Compounds **6a–6x** were assessed for DPPH and ABTS free radical scavenging ability using vitamin C as reference drug. According to the results shown in Table 3, some kojic acid triazol thiosemicarbazide Schiff base derivatives showed more potent DPPH radical scavenging activities than that of reference vitamin C, and most of compounds possessed better ABTS radical scavenging activities than that of reference vitamin C. Compound **6p** exhibited the highest DPPH radical scavenging activity with IC_50_ value of 10.53 ± 0.014 μM, and compound **6w** showed the best ABTS radical scavenging activity with IC_50_ value of 3.03 ± 0.009 μM. Comparing the DPPH free radical scavenging activity, there are the following rules: the activity of compound with ortho position hydroxyl group is better than that of meta position and para position. When R^2^ is hydrogen and R^1^ is halogen atom, the activity increases with the increase of atomic radius, and the activity of compound with 5-halogen atom is lower than that of compound with 4-halogen atom. R^2^ is hydrogen, compounds with alkoxy group possess potent free radical scavenging activity. R^2^ is methyl, the other substituent on the benzene will reduce the activity. For the ABTS free radical scavenging activity, there are the following rules: except for compound **6x**, the activity of compound with methyl (R^2^) is better than that of compound with hydrogen (R^2^). When R^1^ is halogen atom, the activity gradually decreases with the increase of atomic radius, but the activity of compound with 5-halogen atom is better than that of compound with 4-halogen atom. The free radical scavenging activity of compound with alkoxy is better than that of compound with alkyl, and the free radical scavenging activity of compound with para hydroxyl group is better than that of compound with ortho or meta hydroxyl group.

**Table 3.** Antioxidant activity of the target compounds and vitamin C.

Compd	DPPH/IC_50_ (μM) ± SD	ABTS^+^/IC_50_ (μM) ± SD
**6a**	173.20 ± 0.058	43.89 ± 0.034
**6b**	105.40 ± 0.115	63.10 ± 0.042
**6c**	104.70 ± 0.088	72.03 ± 0.084
**6d**	71.43 ± 0.036	91.10 ± 0.033
**6e**	87.47 ± 0.144	8.24 ± 0.048
**6f**	99.60 ± 0.056	34.95 ± 0.061
**6g**	133.80 ± 0.048	52.65 ± 0.109
**6h**	118.40 ± 0.049	68.49 ± 0.091
**6i**	248.10 ± 0.041	61.66 ± 0.091
**6j**	25.60 ± 0.031	6.78 ± 0.082
**6k**	49.17 ± 0.077	9.31 ± 0.022
**6l**	81.67 ± 0.073	40.23 ± 0.071
**6m**	195.00 ± 0.038	36.17 ± 0.018
**6n**	176.20 ± 0.061	44.40 ± 0.068
**6o**	193.90 ± 0.022	17.95 ± 0.048
**6p**	10.53 ± 0.014	3.19 ± 0.034
**6q**	47.05 ± 0.042	6.61 ± 0.054
**6r**	53.54 ± 0.088	4.49 ± 0.103
**6s**	105.80 ± 0.092	11.29 ± 0.033
**6t**	86.30 ± 0.058	3.91 ± 0.064
**6u**	55.70 ± 0.154	5.27 ± 0.078
**6v**	16.27 ± 0.093	3.20 ± 0.091
**6w**	14.44 ± 0.077	3.03 ± 0.009
**6x**	159.20 ± 0.061	19.94 ± 0.081
Vitamin C	27.25 ± 0.015	55.48 ± 0.040

## Conclusions

Twenty-four kojic acid triazol thiosemicarbazide Schiff base derivatives were designed and synthesised, and the chemical structures were characterised by IR, ^1^H NMR, ^13^C NMR and HRMS. The inhibitory activity of the synthesised compounds on tyrosinase was investigated. The results showed that all of the synthesis compounds possessed potent inhibitory activity, and the compound **6w** (IC_50_ = 0.94 ± 0.035 μM) exhibited the best inhibitory effect. For the compounds with halogen atom, with the increase of atomic radius, the inhibitory activity gradually decreases. The inhibitory activity of compounds with alkoxy showed better than that of compounds with alkyl, and compounds with papa hydroxyl group exhibited better inhibitory activity than compounds with ortho or meta hydroxyl group. Compound **6w** is the best inhibitor, and it was selected to explore the inhibition mechanism. Compound **6w** is a mixed inhibitor. The results of fluorescence quenching and UV–visible spectroscopy showed that compound **6w** quenched the fluorescence of tyrosinase by dynamic quenching. FT-IR spectroscopy confirmed that compound **6w** interacted with tyrosinase by changing the amide I band and amide II band in the tyrosinase structure. Molecular docking study further confirmed that compound **6w** was a non-competitive mixed inhibitor, and the kojic acid fragment of the compound could interact with the copper ion active centre and amino acid residues at the same time. Thiosemicarbazide fragment has hydrogen bond interaction with the amino acid residues. The anti-browning effect of compound **6w** is better than that of kojic acid and ascorbic acid. All of the compounds showed certain antioxidant activity, some compounds showed better DPPH free radical scavenging activity than vitamin C, and most of compounds possessed stronger ABTS free radical scavenging activity than vitamin C. Compound **6p** (IC_50_ = 10.53 ± 0.014 μM) exhibited the best DPPH free radical scavenging activity, and the compound **6w** (IC_50_ = 3.03 ± 0.009 μM) had the best ABTS free radical scavenging activity. The free radical scavenging activities of alkoxy substituted compounds exhibited better antioxidant activity than the alkyl substituted compounds. Antioxidant activities of compounds with para hydroxyl group are better than that of compounds with ortho or meta hydroxyl group. The radius of halogen atom and substitution position have obvious influence on the antioxidant activity. All findings together indicated that compound **6w** could be served as the promising candidate for the future development of potent tyrosinase inhibitors, antioxidant and anti-browning agents.

## Data Availability

The data are available within the article.
